# Myeloid-Derived Suppressor Cells (MDSCs) and Obesity-Induced Inflammation in Type 2 Diabetes

**DOI:** 10.3390/diagnostics14212453

**Published:** 2024-11-01

**Authors:** Larisa Ghemiș, Ancuța Goriuc, Bogdan Minea, Gina Eosefina Botnariu, Maria-Alexandra Mârțu, Melissa Ențuc, Daniel Cioloca, Liliana Georgeta Foia

**Affiliations:** 1 Department of Biochemistry, Faculty of Dental Medicine, “Grigore T. Popa” University of Medicine and Pharmacy, 16 Universității Street, 700115 Iasi, Romania; larisa_danaila@yahoo.com (L.G.); ancuta.goriuc@umfiasi.ro (A.G.); bogdan-minea@umfiasi.ro (B.M.); georgeta.foia@umfiasi.ro (L.G.F.); 2 Department of Internal Medicine II, Faculty of Medicine, “Grigore T. Popa” University of Medicine and Pharmacy, 16 Universității Street, 700115 Iasi, Romania; 3 Department of Diabetes, Nutrition and Metabolic Diseases, “St. Spiridon” Emergency County Hospital, 700111 Iasi, Romania; 4 Department of Periodontology, Faculty of Dental Medicine, “Grigore T. Popa” University of Medicine and Pharmacy, 16 Universității Street, 700115 Iasi, Romania; maria-alexandra.martu@umfiasi.ro; 5 Faculty of General Medicine, “Grigore T. Popa” University of Medicine and Pharmacy, 16 Universității Street, 700115 Iasi, Romania; andra-melissa.i.entuc@students.umfiasi.ro; 6 Department of Oro-Dental Prevention, Faculty of Dental Medicine, “Grigore T. Popa” University of Medicine and Pharmacy, 16 Universității Street, 700115 Iasi, Romania; daniel-petru.cioloca@umfiasi.ro

**Keywords:** MDSC, myeloid-derived suppressor cells, inflammation, obesity, insulin resistance, type 2 diabetes

## Abstract

Type 2 diabetes mellitus is a complex metabolic disorder characterized by insulin resistance and, subsequently, decreased insulin secretion. This condition is closely linked to obesity, a major risk factor that boosts the development of chronic systemic inflammation, which, in turn, is recognized for its crucial role in the onset of insulin resistance. Under conditions of obesity, adipose tissue, particularly visceral fat, becomes an active endocrine organ that releases a wide range of pro-inflammatory mediators, including cytokines, chemokines, and adipokines. These mediators, along with cluster of differentiation (CD) markers, contribute to the maintenance of systemic low-grade inflammation, promote cellular signaling and facilitate the infiltration of inflammatory cells into tissues. Emerging studies have indicated the accumulation of a new cell population in the adipose tissue in these conditions, known as myeloid-derived suppressor cells (MDSCs). These cells possess the ability to suppress the immune system, impacting obesity-related chronic inflammation. Given the limited literature addressing the role of MDSCs in the context of type 2 diabetes, this article aims to explore the complex interaction between inflammation, obesity, and MDSC activity. Identifying and understanding the role of these immature cells is essential not only for improving the management of type 2 diabetes but also for the potential development of targeted therapeutic strategies aimed at both glycemic control and the reduction in associated inflammation.

## 1. Introduction

In 2021, there were approximately 537 million people diagnosed with diabetes, and this number is estimated to increase to 643 million by 2030, according to International Diabetes Federation [1]. Of all these cases, over 90% are represented by type 2 diabetes (T2D), making it one of the most common metabolic conditions worldwide [2]. T2D is characterized by insulin resistance, translated by a defective tissue response to physiological levels of insulin, followed by impaired insulin secretion from pancreatic β cells [3,4,5]. These mechanisms contribute subsequently to the development of hyperglycemia, which, over time, favors the occurrence of various diabetes-associated complications, such as macrovascular conditions (coronary artery disease, stroke, and peripheral arterial disorder) and microvascular complications (neuropathy, nephropathy, and diabetic retinopathy) [6].

The etiology of T2D is not yet fully understood [7]. This complex disorder is determined by both genetic and environmental factors, along with socio-economic and demographic conditions [1]. Among these, environmental factors, specifically lifestyle factors hold significant importance. A suboptimal diet, based on insufficient whole-grain intake, excessive consumption of processed meat and refined rice and wheat, along with poor sleep quality, tobacco use, high alcohol consumption, periodontal disease, and obesity, have been correlated with the risk of developing type 2 diabetes [8,9,10,11,12,13,14,15]. Concurrently, obesity enhances the impact of genetic susceptibility and environmental factors on the onset of diabetes. One parameter used to assess obesity is the body mass index (BMI), which classifies individuals with a BMI of 25 to 29.9 as overweight and those with a BMI of 30 or higher as obese [14,15]. Obesity is the primary element involved in the emergence of insulin resistance and, consequently, in the development of T2D [16]. Excessive adiposity triggers a dysregulation of the immune system that leads to the infiltration and activation of immune cells. The chronic inflammation of adipose tissue is a key factor in understanding the mechanisms of the development of insulin resistance and is also a potent target for the treatment of T2D [17]. Over the last decade, myeloid-derived suppressor cells (MDSCs) were identified and described in the obese mice adipose tissue [18], introducing a new population of immune cells with potential involvement in the insulin resistance and type 2 diabetes progress.

The primary aim of this review is to evaluate the role of MDSCs in modulating inflammation associated with obesity-induced type 2 diabetes while presenting current insights and highlighting potential future research directions.

## 2. Type 2 Diabetes and Obesity-Induced Inflammation

Obesity-induced inflammation, also referred to as low-grade inflammation is described as a sub-acute or chronic inflammation of the adipose tissue that impacts other organs and tissues like the liver, skeletal muscle, pancreas, and oral tissues, leading to low-grade systemic inflammation [19,20,21,22]. Beyond its role in energy storage, adipose tissue is recognized for its multiple functions [23]. Increased fat directly affects adipose cells and their secreted factors [23,24]. Under these conditions, the elevated synthesis and secretion of adipokines and cytokines settle the adipose tissue as the largest endocrine organ [25].

Two important enzymes involved in glucose metabolism and, consequently, in adipose tissue accumulation are pancreatic alpha-amylase and intestinal alpha-glucosidase. Alpha-amylase hydrolyzes carbohydrates by breaking down α-1,4-glycosidic bonds, resulting in the formation of oligosaccharides, which are further converted into glucose by alpha-glucosidase. Inhibiting both enzymes can effectively delay carbohydrate digestion and the intestinal absorption of glucose, thereby reducing postprandial hyperglycemia [26,27]. Another important protein, leptin, is a hormone produced by adipocytes that regulates energy homeostasis and food intake. It plays an important role in the insulin–glucose axis and insulin responsiveness [28]. Recent studies indicate a positive correlation between serum leptin levels and insulin resistance, relatively high leptin levels being correlated with lower insulin sensitivity and hyperinsulinemia [29,30]. Leptin secretion is also correlated with adiposity, with obese individuals presenting elevated levels of leptin. However, the fat accumulation in their case is not influenced due to leptin resistance [31]. Leptin is also an important component of hypothalamic leptin–melanocortin signaling pathway. Genetic disruptions in this pathway are responsible for most monogenic forms of severe obesity in both mice and humans [32].

Leptin plays an important role in triggering inflammation, bone homeostasis regulation and oxidative stress [33]. One important function resides in lowering the mitochondrial lipid peroxidation products and thus mitochondrial function improvements [34,35]. Mitochondrial dysfunction is a key mechanism involved in the relationship between obesity and metabolic complications, leading to insulin resistance by inducing interleukin 1β (IL-1β) secretion through the activation of NRP3 inflammasome [34,36,37]. Obesity-related high leptin expression and insulin resistance are also correlated with the prognosis of type 2 diabetes [33]. In his study, Senkus analyzes the adipose tissue dysfunction and the adiponectin/leptin ratio, and the results point out that the adiponectin/leptin ratio is strongly associated with adiposity, being improved by weight loss [38]. Being inversely correlated with obesity and visceral adiposity, adiponectin supports interleukin 10 (IL-10) expression and inhibits the nuclear factor kappa light-chain-enhancer of activated B cells (NFκB), influencing interferon gamma (IFNγ) production in monocyte-derived cells [39]. Decreased adiponectin levels can be correlated with an increased risk of type 2 diabetes in patients without risk factors [40].

In obese conditions, inflamed adipocytes up-regulate cytokine secretion, including tumor necrosis factor-α (TNF-α), interleukin 6 (IL-6), interleukin 1β (IL-1β), and monocyte chemo-attractant protein-1 (MCP-1/CCL2) [41]. High levels of these mediators are also found in patients with type 2 diabetes, subsequently triggering impaired insulin sensitivity and glucose homeostasis [42,43]. Apart from high levels of pro-inflammatory cytokines, there are other factors that can contribute to the development of insulin resistance, such as the overproduction of reactive oxygen species (ROS), glucolipotoxicity, and the activation of transcriptional-mediated pathways [44].

### 2.1. Inflammation and Impaired Insulin Signaling

Obesity-induced lipid accumulation within the adipose cells triggers the activation of inflammatory pathways. Cytokines can also activate these pathways, finally leading to more pro-inflammatory cytokine production [45]. For instance, TNF-α, Il-1β, and Il-6 can activate the IκB kinase-β/nuclear factor-κB (IKK-β/NF-κB) and c-Jun amino-terminal kinase (JNK) pathways [3,43]. The NF-κB transcription factor induces the expression of genes that encode cytokines, chemokines, and other pro-inflammatory molecules, making it a crucial mediator of inflammatory responses [46]. This activation plays a significant role in the development of diabetes and its associated chronic complications [47]. The primary mechanism for the activation of NF-κB consists of the phosphorylation of IκB regulator by IκB kinases (IKKS) [48], in response to the binding of cytokines to receptors such as TNF receptor (TNFR) and pattern-recognition receptor (PRR) [46]. Kinases such as IKKβ and JNK can induce the serine phosphorylation of IRS-1, a pivotal component of the insulin signaling pathway associated with insulin sensitivity, leading to insulin resistance and impaired glucose uptake in muscle cells and adipocytes [49].

The NF-κB-mediated pathway directly participates in the activation of the NLRP3 family (which encodes NOD—nucleotide-binding oligomerization domain, LRR—leucine-rich repeat, and pyrin domain-containing protein 3) and the accompanying inflammasome, being involved in the advancement of insulin resistance associated with obesity and diabetes [50,51,52]. In a study using the rat as an experimental model, in H9c2 cells exposed to high concentrations of glucose, the NF-κB-mediated pathway leads to the activation of the NLRP3 inflammasome [50,53]. In obesity, macrophages and cells of the myeloid lineage are the main cells that express NLRP3 [51]. Furthermore, NLRP3 inflammasome is also activated by ROS due to increased levels of saturated fatty acids and hyperglycemia, subsequently activating caspase-1, resulting in the secretion of IL-1β and IL-18, driving systemic inflammation, impairing pancreatic β-cell function, and inducing insulin resistance [52,54,55].

### 2.2. Adipose Tissue Inflammation and Immune Cells

The infiltration of adipose tissue by immune cells is driven by increased cytokine and chemokine production, maintaining local and systemic chronic inflammation [56]. MCP-1 is secreted in high amounts by hypertrophied adipocytes and acts as a chemoattractant for inflammatory cells [24]. Macrophages are the most abundant cell type resident in the adipose tissue, while the phenotype of infiltrated cells is strongly influenced by obesity [9]. Moreover, although, in lean adipose tissues, the main cellular types are T regs, Th2, and M2 macrophages, the adipose tissue of obese subjects is infiltrated by activated macrophages, especially of the M1 phenotype, Th17, Th1, CD8+ T lymphocytes, and dendritic cells [17,57,58].

Systemic inflammation linked to obesity also influences the immune cells in adipose deposits [59]. An accumulation of a new group of cells with immature characteristics, named myeloid-derived suppressor cells (MDSCs) was identified in white adipose tissue during obesity, over ten years ago [18]. MDSCs can regulate and suppress the immune responses that impact adipose tissue inflammation and consequently insulin resistance [59,60].

## 3. Myeloid-Derived Suppressor Cells (MDSCs) and Inflammation

### 3.1. MDSC Origin and Phenotypes

The terminology of myeloid-derived suppressor cells (MDSCs) was settled about 16 years ago [61]. MDSCs refers to a heterogeneous family of immature myeloid cells, originating from the bone marrow, with an important immunosuppressive role on T cells and other immune cells [61,62]. There are two main subpopulations of MDSCs, granulocytic MDSCs (G-MDSCs, also known as polymorphonuclear MDSCs, PMN-MDSCs), with a neutrophil-like morphology, and monocytic MDSCs (M-MDSCs), with a morphology that resembles monocytes [62]. In addition to these two, there is a more immature subpopulation of MDSCs called early-stage MDSCs (E-MDSCs) that does not express specific markers for granulocytes or monocytes [63].

From the perspective of surface phenotype in humans, G-MDSCs are cells with CD11b^+^CD14^+^CD15^+^ or CD11b^+^CD14^+^CD66b^+^ phenotypes, while M-MDSCs have the CD11b^+^CD14^+^HLADR^−/low^CD15^−^ phenotype. Cells belonging to the E-MDSC population do not express either CD14 or CD15 and are defined as Lin^−^ HLADR^−^CD33^+^ [61,64]. Table 1 provides an overview of the MDSC subsets and their cell-surface markers both in humans and in mice. The activity of these cells was intensively studied in tumor pathology, as well as infectious and autoimmune diseases. MDSCs exhibit immunoregulatory properties manifested in all pathologies involving the immune system [62].

### 3.2. The Role of MDSCs in Immune Regulation

Regardless of the MDSC subset, they suppress T cells using a variety of mechanisms. MDSCs inhibit NK cells and induce regulatory T cells through the secretion of cytokines and via direct cell–cell contact. The primary factor influencing these complex interactions is the microenvironment within the tissue where they occur [67].

MSDCs have been mostly studied in the tumor microenvironment, where it was observed that through the production of nitric oxide (NO), ROS, IL-10, and TGF-β, they are able to stimulate Tregs expansion, and through cysteine and arginine secretion inhibition, they restrict the function of effector T cells [68]. Arginine deficiency is triggered through MDSC-induced arginase 1 secretion, which induces a decrease in this aminoacid absorption, thus leading to the inhibition of the expansion of T cells by ceasing their cell cycle. The blockade of T cell activation can occur consecutively from the dysregulation of the T cell receptor TCR chain due to decreased cysteine availability [69].

Another important mechanism by which MDSCs modulate/suppress the immune system is the secretion of inducible nitric oxide synthetase (iNOS) and NADPH oxidase (NOX2), which consecutively leads to the generation of NO and ROS [70,71,72].

## 4. MDSCs and Adipose Tissue Inflammation in T2D

### 4.1. Recruitment of MDSCs in Adipose Tissue in T2D

Friedrich et al. report an elevated number of MDSCs in peripheral blood of obese individuals, associated with increased adiposity, HbA1c, and inflammation [73]. However, the accumulation of MDSCs enhances insulin response, while the depletion of MDSCs results in reduced glucose tolerance and insulin resistance [18,74].

Myelopoiesis is induced by colony-stimulating factors (CSFs) that include a group of cytokines such as macrophage colony-stimulating factor (M-CSF) and granulocyte/macrophage colony-stimulating factor (GM-CSF). MDSCs can also be induced from monocytes by GM-CSF in vitro [75]. Myeloid cells express pattern recognition receptors (PRRs), including Toll-like receptors (TLRs). TLR ligation activates myeloid cells classically, and it results in the rapid mobilization of neutrophils and monocytes from bone marrow [76]. TLRs can also be activated by damage-associated molecular patterns (DAMPs) and hyperglycemia present in T2D [77]. The phenotype and morphology of these generated cells are immature, with an increased production of ROS, nitric oxide (NO), and anti-inflammatory cytokines along with an increased expression of arginase and a weak phagocytic activity [76]. MDSC expansion in the bone marrow is also induced by the increased JNK and IKKβ signaling pathways in diabetes [78].

Chemokines and cytokines present in chronic inflammation can drive MDSCs from peripheral blood and bone marrow to the inflammation site. In tumors, there is evidence that CC-chemokine ligand 2 (CCL2) regulates the accumulation of M-MDSCs, while CC-chemokine ligand 5 (CCL5) is responsible of PMN-MDSC control [75]. A meta-analysis concluded that the concentration of CCL2 and CCL5 were significantly higher in type 2 diabetic patients when compared to controls [79]. Moreover, it had been shown that adipocytes secrete CCL2 to promote the migration of myeloid cells into tissue [80]. Tumor progression and obesity are closely related and, in trying to understand the mechanism behind their relationship Turbitt, proved the accumulation of MDSCs in the tumor, spleen, and adipose tissue of tumor-bearing mice. Moreover, the increased adiposity was correlated with the accumulation of Gr1+Cd11b+ cells [81]. Additionally, it was shown that genetically obese (ob/ob) mice have an increased infiltration of Gr1+Cd11b+ cells in adipose tissue in the absence of a tumor [18,81].

**Figure 1:**
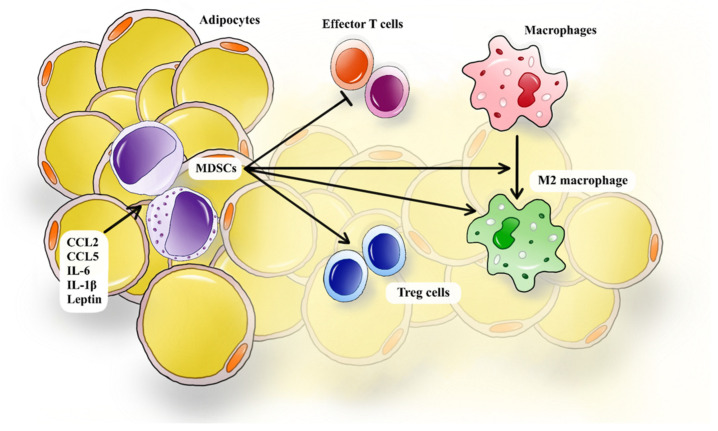
MDSC roles in adipose tissue inflammation in T2D. MDSCs are recruited in adipose tissue by pro-inflammatory molecules: CCL2, CCL5, IL-6, IL-1β, and leptin. MDSCs suppress effector T cell activity, may induce Treg cells, promote the differentiation, and enhance the stability of M2 macrophages. MDSCs, myeloid-derived suppressor cells; T2D, type 2 diabetes; CCL2, CC-chemokine ligand 2; CCL5, CC-chemokine ligand 5; IL-6, interleukin 6; Treg cells, regulatory T cells.

Cytokines can also induce MDSC chemotaxis in different tissues. Giannan’s study proved that the up-regulation of IL-6 in ob/ob mice was positively correlated with the presence of MDSCs in the tumor microenvironment in ovarian cancer [82]. Similar, IL-1β secreted by tumoral cells into the tumor microenvironment was shown to drive the accumulation of MDSCs with an increased ability to suppress T cells [83]. Since IL-1β and IL-6 levels are increased in type 2 diabetes and are primarily released from adipose tissue and visceral fat, it can be hypothesized that these pro-inflammatory cytokines may support the accumulation of MDSCs in adipose tissue [42,84]. Similarly, as leptin levels are influenced by obesity-induced inflammation, over-expressed leptin was positively correlated with the accumulation of MDSCs in mice on a high-fat diet [85]. Correlating these data, we can assume that CCL2, CCL5, IL-6, IL-1β, and leptin can play a significant role in MDSC recruitment in the adipose tissue of patients with type 2 diabetes (Figure 1).

### 4.2. The Interplay Between MDSCs and Immune Cells in T2D

#### 4.2.1. MDSCs and Macrophages

Macrophages infiltrated in adipose tissue, also called adipose tissue macrophages (ATM), represent a heterogeneous population consisting of two macrophage populations, M1 and M2 [86]. The differentiation between these populations is determined by their type of activation as M1 is classically activated, while M2 is alternatively activated. Additionally, their roles in adipose tissue inflammation further distinguish them [87]. Macrophages adapt their function according to environmental stimuli through surface receptors [88]. M1 macrophages, often named pro-inflammatory macrophages and induced by TLR binding or Receptor for Advanced Glycation End Products (RAGE), or secondary to the action of IFN-γ, express high levels of pro-inflammatory cytokines such as TNF-α, IL-1β, IL-6, and IL-23 [87,89]. On the other hand, M2 macrophages secrete anti-inflammatory cytokines like IL-10 and IL-4 and exhibit an increased expression of arginase-1 [89,90].

In the adipose tissue of healthy individuals, M2 macrophages represent over 90% of the total ATM [86]. However, with increasing adiposity, there is a transformation of ATM into the pro-inflammatory M1 phenotype, promoting chronic low-grade inflammation of the adipose tissue [90]. M2 macrophages can modulate the Th1/Th2 and Th17/Treg balance, promoting the differentiation of Th2 and Treg subsets, thereby ameliorating inflammation in renal neuropathy [91].

In cancer patients, MDSCs induce macrophage polarization toward M2 phenotype by down-regulating IL-12 and up-regulating IL-10 production [92]. In obese subjects, peripheral tissues are highly enriched with Gr1+CD11b+ cells; these immature myeloid cells can induce macrophage differentiation into M2 macrophages, promoting insulin sensitivity [18]. MDSCs can also enhance the stability of the “M2”-like phenotype of ATMs, with their depletion being associated with an increase in “M1”-like ATMs [93]. In addition, MDSCs and monocytes can also differentiate into the alternative activated M2 macrophages through the activation of transcriptional factors. One of the transcription factors with this essential role is the peroxisome proliferator-activated receptor γ (PPARγ) [94]. White adipose tissue expresses PPARγ, which, in turn, can control adipogenesis; hence, it can also impact insulin sensitivity through its expression in the muscle and liver [95].

#### 4.2.2. MDSCs and Effector T Cells

T lymphocytes respond to cytokine and chemokine signals by infiltrating the obese adipose tissue. They are involved in the secretion of pro-inflammatory cytokines and can also promote the activation of macrophages [96]. The T cell population consists of CD4+ and CD8+ T cells. Depending on the secreted cytokines, CD4+ T cells are divided into T helper type 1 cells (Th1 which secrete IFNγ), T helper type 2 cells (Th2 which secrete mainly IL4 and IL13), and T helper 17 cells (Th17 which secrete IL17) [97].

CD4+ T cells are increased in the adipose tissue of obese mice and are linked to the development of insulin resistance [98,99]. The persistent inflammation in T2D is intricately connected to the Th1 and Th2 immune responses [98]. The Th1/Th2 ratio is influenced by the cytokine environment and impaired Th1/Th2 responses can lead to the development of T2D and its associated complications [97,100]. Elevated levels of Gr1+CD11b+ cells in db/db mice (with defective leptin signaling) suppress CD4+ T cells and hinder the function of CD8+ T cells through the release of IFN-γ and iNOS [18,74]. In cell culture, increased levels of IL-10 and TGF-β are associated with inhibitory effects of MDSCs on CD4+ T cells [101]. In a study on high-fat diet (HFD) mice, increased MDSC levels in their blood limits the activation of CD8+ T cells [85]. Additionally, MDSCs suppress the proliferation of CD8+ T cells and even induce their apoptosis [18]. Thus, it would be pertinent to claim that MDSCs can prevent the development of T2D by limiting CD4+ and CD8+ T cells activity.

#### 4.2.3. MDSCs and Tregs

Another subgroup of CD4+ T cells is represented by regulatory T cells (Tregs), which has a primary role to regulate the activity of other effector T cells and prevent reactivity towards autoantigens [102]. The nuclear transcription factor forehead box protein P3 (FOXP3) modulates the function of Tregs and is involved in inducing their immunosuppressive functions [103]. Tregs can produce anti-inflammatory cytokines such as TGFβ and IL10, while Th17 cells disclose pro-inflammatory properties through their cytokine production. The Th17/Treg ratio has a major contribution to the chronic inflammation associated with obesity and can be influenced by IL6, which triggers the Th17 subset [102,104]. Some studies proved that the presence of FOXP3+ Tregs was associated with improved insulin sensitivity, while their depletion in the visceral fat of obese mice was correlated with insulin resistance [105,106,107]. However, Zhu found that, in type 2 diabetes, FOXP3+ Tregs can present a dysregulated function. They described that the frequency of FOXP3+ Tregs, which express higher levels of IL-17, was correlated with HbA1c and body mass index [105].

Low-grade chronic inflammation is a key factor linking obesity and type 2 diabetes. An increased accumulation of M-MDSCs (CD33^+^CD11b^+^CD14^+^HLADR^low/−^) in the peripheral blood of obese patients was reported compared to lean patients, which was correlated with impaired T cell functions [108]. MDSCs can suppress T cell activity by increasing the expression of programmed cell death protein-ligand 1 (PD-L1) on their cell surface. Hypoxia, IL-10, and VEGF (vascular endothelial growth factor) are three important modulators of PD-L1 expression in MDSCs [67,72]. It is hypothesized that leptin may increase the suppressive activity of MDSCs by increasing PD-L1 expression on MDSCs [109].

Programmed cell death protein 1 (PD-1) is the receptor of PD-L1 which is mainly expressed by T cells, macrophages, and dendritic cells [72,110]. The PD-1/PD-L2 pathway may influence the peripheral immune tolerance of T cells and, consequently, the development of type 2 diabetes [110,111]. Decreased levels of PD-1+ Tregs and PD-L2+ M-MDSCs were associated with increased T cell activation, while PD-1+ Tregs were negatively correlated with waist circumference, fasting insulin level, and HbA1c [111]. Compared with healthy subjects, lower expressions of PD-1 on monocytes, CD4+ T cells, and CD8+ T cells were identified in patients with type 2 diabetes [110]. Therefore, PD-1 expression on immune cells and M-MDSCs and Tregs may be crucial mediators of chronic inflammation in type 2 diabetes and may be involved in the progression of this condition.

## 5. MDSCs and Diabetic Complications

Diabetic nephropathy (DN), also known as diabetic kidney disease, is a common chronic complication of type 1 or 2 diabetes that leads to end-stage renal disease [112]. Islam’s study finds an increased proportion of PMN-MDSCs in T2D patients (96% of the total MDSCs), which can be correlated with renal progression. Hyperglycemic conditions augment the anti-inflammatory abilities of MDSCs; however, their function is surpassed by the pro-inflammatory molecules, making them insufficient for maintaining kidney function in type 2 DN [113].

Glomerulosclerosis is defined by the accumulation of extracellular matrix proteins in the mesangial interstitial space, leading to fibrosis and contributing to the development of DN. In vitro-induced MDSCs by IL-1β, IL-6, and GM-CSF express higher immunoregulatory functions and can decrease the production of fibronectin, leading to the improvement in renal fibrosis [114]. Another study conducted by Li proves the capacity of cytokine-induced MDSCs to decrease fibronectin production in renal glomerulus, improving proteinuria and renal function in diabetic mice [115]. When considering immunotherapy for the prevention of T2D and its complications, MDSCs could be part of a therapeutic strategy for DN, but further studies are required.

Diabetic ocular complications include diabetic retinopathy and ocular surface complications such as corneal disorders. Qin shows in his study that the immune microenvironment is altered in diabetic corneas. Surprisingly, an increased number of MDSCs and gamma delta (γδ) T cells are correlated with a decrease in Tregs and CD103+CD8+ tissue-resident memory (TRM) cells [116]. Additionally, Li’s study indicates that MDSC, Th17 cells, and activated B cells are key immune cells involved in the process of immune infiltration both in diabetic retinopathy and atherosclerosis (AS) [117]. A high proportion of MDSCs in retinal tissue can contribute to retinal vascular inflammation and angiogenesis, processes implicated in diabetic retinopathy and its associated complications [118,119]. However, the effects on MDSCs might depend on the disease phase. In the acute phase of autoimmune uveoretinitis, MDSCs have beneficial roles in reducing disease severity. In contrast, during the chronic phase, their positive effects are outweighed by their role in promoting angiogenesis and tissue damage [120]. Recent research has shown that the cannabidiol-induced up-regulation of MDSCs exhibits anti-inflammatory and anti-angiogenic effects in suture-induced corneal neovascularization [121].

The progression of T2D is often accompanied by impaired wound healing, with diabetic ulcers being a common complication. In diabetic mice and under hyperglycemic conditions, MDSC function becomes dysfunctional, resulting in hindered wound repair. However, the suppression of the mTOR (mammalian target of rapamycin) pathway can counteract the glucose-induced dysfunction in MDSCs, thereby accelerating wound healing and highlighting the detrimental role of MDSCs in this context [122]. On the other hand, the use of KLF4 (Kruppel-like factor 4) enhances diabetic wound healing by increasing the number of MDSCs. Additionally, MDSCs were shown to effectively suppress Th17 differentiation and IL-17A production in vitro [82]. Thus, MDSCs can exhibit a dual role in diabetic wound healing: impaired function due to hyperglycemia leads to delayed repair, while modulation through mTOR inhibition or KLF4 treatment can restore their beneficial effects. This underscores the potential of targeted therapies to improve wound healing, though further research is needed to fully understand the complex role of MDSCs in diabetic complications.

The condition of patients with chronic complications of T2D can be worsened by the presence of other comorbidities, such as additional chronic or acute illnesses. For example, in these patients, SARS-CoV-2 infection can be more severe, accompanied by a cytokine storm and β-cell damage, leading to a worse prognosis compared to individuals without T2D [123,124,125]. A recent study presents a correlation between the diagnosis of COVID-19 and T2D among children and adolescents aged 10 to 19, suggesting that SARS-CoV-2 may be considered a risk factor for the development of diabetes [126]. A key element driving this acute infection is the ability of the SARS-CoV-2 Spike receptor-binding domain (RBD) to bind to epitopes present on various clusters of differentiation (CDs), such as CD147 [127]. The presence of the transmembrane protein CD147 is associated with increased blood glucose levels in COVID-19 patients and is positively correlated with BMI, obesity, advanced glycation end products (AGEs), and matrix metalloproteinase (MMP) levels [128,129,130]. Additionally, CD147 is correlated with the recruitment of MDSCs via the RSK2/AP-1 pathway, and elevated levels of M-MDSCs are associated with a higher risk of infections and tumor development in T2D patients [131,132]. The inhibition of CD147 effectively reduces SARS-CoV-2 amplification and may represent a new approach for treating hyperglycemia and T2D, though further studies are required [130].

## 6. MDSCs and Anti-Diabetic Therapy

Metformin is an oral hypo-glycemic drug primarily used in the treatment of type 2 diabetes for its beneficial effects in reducing hepatic glucose synthesis and increasing peripheral tissue sensitivity to insulin, mainly through the stimulation of AMPK enzymes (AMP-dependent kinase) [133]. Additionally, metformin strongly activates the immune system and has garnered interest in the past decades for its potential benefits in neoplastic pathology, as it can reduce the risk for several types of cancers [134,135]. Some of these effects are attributed to their effects on tumor-infiltrating immune cells, particularly MDSCs. Metformin administration can directly reduce the number of MDSCs and Tregs in the tumor microenvironment and inhibit their immunosuppressive effects, thereby slowing tumor progression [134]. Hatae reports that treatment with Met+Rap, pretreated CAR-T cells (AMPK activator metformin and the mTOR inhibitor rapamycin, pretreated chimeric antigen receptor T cell), significantly extended the survival of mice with intracerebral gliomas while decreasing the intratumoral MDSCs [136,137]. Moreover, the anti-tumor effect of metformin is further evidenced by its ability to reduce the accumulation of PMN-MDSCs in diabetic patients with esophageal squamous cell carcinoma [138].

These findings suggest that metformin therapy can modulate the tumor microenvironment by decreasing MDSC accumulation, thus improving the prognosis of patients with various tumor types and stages. Therefore, metformin therapy can even influence the inflammatory microenvironment, modifying MDSC action. This fact must be taken into consideration when the effects and properties of this type of cell are studied to obtain precise results and exclude possible interferences.

## 7. Conclusions

In obesity-induced type 2 diabetes, pro-inflammatory molecules like IL-1β, IL-6, CCL2, CCL5, and leptin play key roles in the accumulation of MDSCs in adipose tissue. These MDSCs influence both the composition and activity of immune cells, promoting the M2 phenotype in macrophages, which enhances insulin sensitivity. Additionally, MDSCs can suppress the activity of CD4+ and CD8+ T cells, thereby preventing the development of type 2 diabetes. While MDSCs are known to induce Tregs in tumor microenvironment, this characteristic has not yet been fully established in the context of chronic low-grade inflammation of adipose tissue associated with type 2 diabetes. Regarding the chronic complications of type 2 diabetes, MDSCs were shown to reduce fibronectin production, improving fibrosis and renal function in diabetic nephropathy. However, their role in diabetic retinopathy remains unclear, with studies providing inconclusive results about whether their impact is beneficial or harmful. Targeting MDSCs for therapeutic purposes shows the most promise in diabetic wound healing, where two therapeutic agents have effectively harnessed the positive properties of MDSCs. All in all, further research is essential to unravel the precise mechanisms through which MDSCs influence diabetes pathology and to develop targeted interventions that can leverage their role in disease management.

## Figures and Tables

**Table 1:** Cell-surface markers of MDSCs.

Subset	Phenotype	Cell Type	Reference
G-MDSCs	CD11b^+^CD14^−^CD15^+^ or CD11b^+^CD14^−^CD66b^+^	human	[61,65]
	CD11b^+^Ly6G^+^Ly6C^lo^	mice	[61,65]
M-MDSCs	CD11b^+^CD14^+^HLA-DR^−/low^CD15^−^	human	[61,65]
	CD11b^+^Ly6G^−^Ly6C^hi^	mice	[61,65]
E-MDSCs	Lin^−^ HLADR^−^CD33^+^	Human	[66]
	-	mice	

MDSCs, myeloid-derived suppressor cells; G-MDSCs, granulocytic myeloid-derived suppressor cells; M-MDSCs, monocytic myeloid-derived suppressor cells; E-MDSC, early-stage myeloid-derived suppressor cells.

## Data Availability

Not applicable.
